# Supplementary material for: Chromothripsis during telomere crisis is independent of NHEJ, and consistent with a replicative origin
Source: Genome Res. 2019 May;29(5):737–49. doi: 10.1101/gr.240705.118 (PMC6499312; doi:10.1101/gr.240705.118)
Supplement: Supplemental Material [file supp_gr.240705.118_Supplemental_file_1.zip › contigs/annotated_contigs/DB56/contig.2.DB56_length_675_mean_cov_4.76.docx]

**DB56_length_675_mean_cov_4.76**

```
CACTATACTATATGTACTTCCATACTTAGAAAAACAACCAGAGGAGTGCACACCAGGTTATTAATAGTAATTATCTCTGGAGGTAAGAT
 >chr7:138203030-138203336 + E=2e-172
TATTTCTGAAATGTTTGGATTTTGCCTTTAATTATTAGTAAGATAACTGCGGTTTACTATTTAACCATATTTATTCATATTCTGATTTT

TTCACTTAACATTATGACATAAACATTTTCCAGTTTTATCCCCACACTGGAAAACTAGAGTGTAATAATGAAAACATTATTATTTCTGT

GTTAATGGGTATTCTCTGTTAGGATTTTTACAAAAGTGG|TTATCTGCTGACCTTCTCTCCACTATTATCCTATGACCCTGCCACATCC
                                         >chr7:139152865-139153207 + E=5e-185
CCCTCTCCGAGAAACACCCAAGAATGATCAATAAATACTAAGGGAACTCAGAGGCTGGCGGGATCCTCTATATGCTGAACGCTGGTCCC

CTGGGCCCCCTTATTTCTTTCTCTATACTTTGTCTCTGTGTCTTTTTCTTTTCCAAGTCTCTCGTTCCACCTAACAAGAAAGACCCACA

GGTGTGGAGGGGCAACCCACCCCTTCAATTCCTGCCTTAACTGATGACATTACTTTGGGAAAGTCCTTTTCCTGGATCATAAGCTCCCC

AACTGAGCACATTGTGACCCTTGCCC|AGGCCGGCCAGGGGGCAACCCTCTTCG
```
